# The genome sequence of the English holly, *Ilex aquifolium *L. (Aquifoliaceae)

**DOI:** 10.12688/wellcomeopenres.20748.1

**Published:** 2024-01-03

**Authors:** Maarten J. M. Christenhusz, Michael F. Fay, Royal Botanic Gardens Kew Genome Acquisition Lab, Plant Genome Sizing collective, Darwin Tree of Life Barcoding collective, Wellcome Sanger Institute Tree of Life Management, Samples and Laboratory team, Wellcome Sanger Institute Scientific Operations: Sequencing Operations, Wellcome Sanger Institute Tree of Life Core Informatics team, Tree of Life Core Informatics collective, Darwin Tree of Life Consortium

**Affiliations:** 1 Royal Botanic Gardens Kew, Richmond, England, UK; 2 Curtin University, Perth, Western Australia, Australia

**Keywords:** Ilex aquifolium, English holly, genome sequence, chromosomal, Aquifoliales

## Abstract

We present a genome assembly from an individual *Ilex aquifolium* (the English holly; Eudicot; Magnoliopsida; Aquifoliales; Aquifoliaceae). The genome sequence is 800.0 megabases in span. Most of the assembly is scaffolded into 20 chromosomal pseudomolecules. The assembled mitochondrial and plastid genomes have lengths of 538.43 kilobases and 157.52 kilobases in length, respectively.

## Species taxonomy

Eukaryota; Viridiplantae; Streptophyta; Streptophytina; Embryophyta; Tracheophyta; Euphyllophyta; Spermatophyta; Magnoliopsida; Mesangiospermae; eudicotyledons; Gunneridae; Pentapetalae; asterids; campanulids; Aquifoliales; Aquifoliaceae; *Ilex*; *Ilex aquifolium* L., 1753 (NCBI:txid4298).

## Background

Common holly, *Ilex aquifolium* L., is an evergreen, dioecious shrub or small tree, found across western and southern Europe, east to Germany and Romania and south to Morocco, but it has been planted and naturalised from cultivation across Europe and in Tasmania, New Zealand and western North America ( POWO, 2023).

It is one of the few evergreen hardwood trees in Britain, where it usually grows in shaded deciduous forests under oak or beech. It most frequently grows as a shrub up to 3 m tall, but occasionally trees of up to 23 m are found (e.g. Peterken & Lloyd, 1967). It is long-lived, with some individuals known to be more than 300 years old ( De Cleene & Lejeune, 2000); in Britain, some trees have been shown to be more than 250 years old ( Peterken & Lloyd, 1967). The evergreen leaves are also long-lived and can stay alive for up to five years. In the leaf axils white, four-parted unisexual flowers appear. These are sweet-scented and are pollinated by bees, wasps, flies and small butterflies. On female plants the flowers develop into red drupes with the persistent stigma on top. Seeds are dispersed in late winter by birds or rodents. The species is adaptable to different situations and can quickly invade clear-cuts or forest margins, but holly is damaged by fire, grazing and severe frost, which can delimit its distribution. It is also the host for the larvae of the holly leaf miner, *Phytomyza ilicis* ( Curtis, 1846), which causes yellow patches on the leaves.

The evergreen leaves of holly have spinose margins in the lower part of the plant, whereas they become smooth and spine-less in the upper part. This phenotypic plasticity is a response to grazing. Epigenetic variation caused by changes in DNA methylation are responsible for phenotypic plasticity in *Ilex aquifolium* ( Herrera & Bazaga, 2013).

In the Mediterranean, holly was associated with the Roman festival Saturnalia (17 December), where branches were given to friends as a good luck charm. Early usage of holly in the Christian church is probably derived from this usage ( De Cleene & Lejeune, 2000). In Germanic and Celtic druidic traditions, it was associated with winter solstice festivities, because it was one of the few evergreen trees in the forest and therefore considered sacred. These associations with winter remained and were later merged with Christmas, so holly is now commonly featured in Christmas decorations with other evergreen species including *Viscum album* L. (mistletoe) and *Hedera helix* L. (ivy). With the latter species, holly is the focus of the traditional Christmas carol “The holly and the ivy”. These evergreen plants are symbols of life in northern peoples, especially in Britain. It was seen as a protective plant and hence it was frequently planted near churches and monasteries, often with other evergreen species including *Taxus baccata* L. (yew). Many apotropaic powers are attributed to holly ( Grigson, 1955).

It also has an association with Easter, especially in Germany and Switzerland, where the spiny branches were used for chastisement and were also burned to celebrate the resurrection of Christ. With its blood-red berries and spiny leaves, it is also a symbol of the crown of thorns ( De Cleene & Lejeune, 2000).

The wood was traditionally used to make Great Highland bagpipes in Scotland, although this usage was abandoned in favour of cheaper tropical hardwoods that became available during the 19th century ( Dickson, 2009). The wood is easily worked and is often used for handles and cabinetry. Bark was once used to make a tar-like substance to catch birds ( Dodoens, 1554). The same book also describes a treatment for abdominal pain; however, as holly is poisonous, it should not be consumed.

The name holly comes from the Old English ‘holen’, probably derived from a Proto-Indo-European root for prick; holly and its Celtic equivalents (‘kelen’ in Cornish; ‘celyn’ in Welsh) are frequent placename elements. *Ilex* is the Latin word for the holm oak ( *Quercus ilex* L.), which has similar prickly leaves, and *aquifolium* is derived from Latin ‘acus’, needle, and ‘folium’, leaf. All these terms refer to the prickly leaves.

We sampled a female individual from a native stand at Petersham Common in Surrey, where it grows in mixed woodland with *Acer campestre* L., *Carpinus betulus* L. and *Quercus robur* L. As with the genome of the Chinese *Ilex micrococca* Maxim. ( Yao *et al.*, 2022), the genome of *Ilex aquifolium* will be equally useful for population genetic studies, especially related to studying phenotypic variation in glacial refuges across the range of this species ( Mihali *et al.*, 2022). We hope that this high-quality genome will be useful for furthering the studies on population genetics, epigenetics and domestication in Aquifoliaceae.

## Genome sequence report

**Figure 1.**
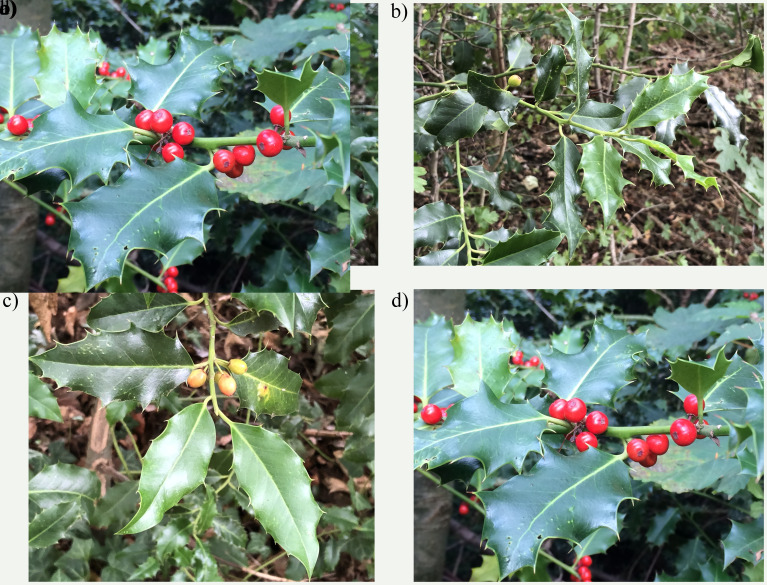
Photographs of the *Ilex aquifolium* (drIleAqui2) specimen used for genome sequencing.

The genome was sequenced from a specimen of *Ilex aquifolium* ( Figure 1) collected from Petersham Common, Richmond, Surrey, UK (51.45, –0.30). Using flow cytometry, the genome size (1C-value) was estimated to be 1.04 pg, equivalent to 1,010 Mb. A total of 36-fold coverage in Pacific Biosciences single-molecule HiFi long reads was generated. Primary assembly contigs were scaffolded with chromosome conformation Hi-C data. Manual assembly curation corrected 16 missing joins or mis-joins and removed 7 haplotypic duplications, reducing the assembly length by 0.48% and increasing the scaffold number by 0.95%, while decreasing the scaffold N50 by 0.84%.

**Figure 2.**
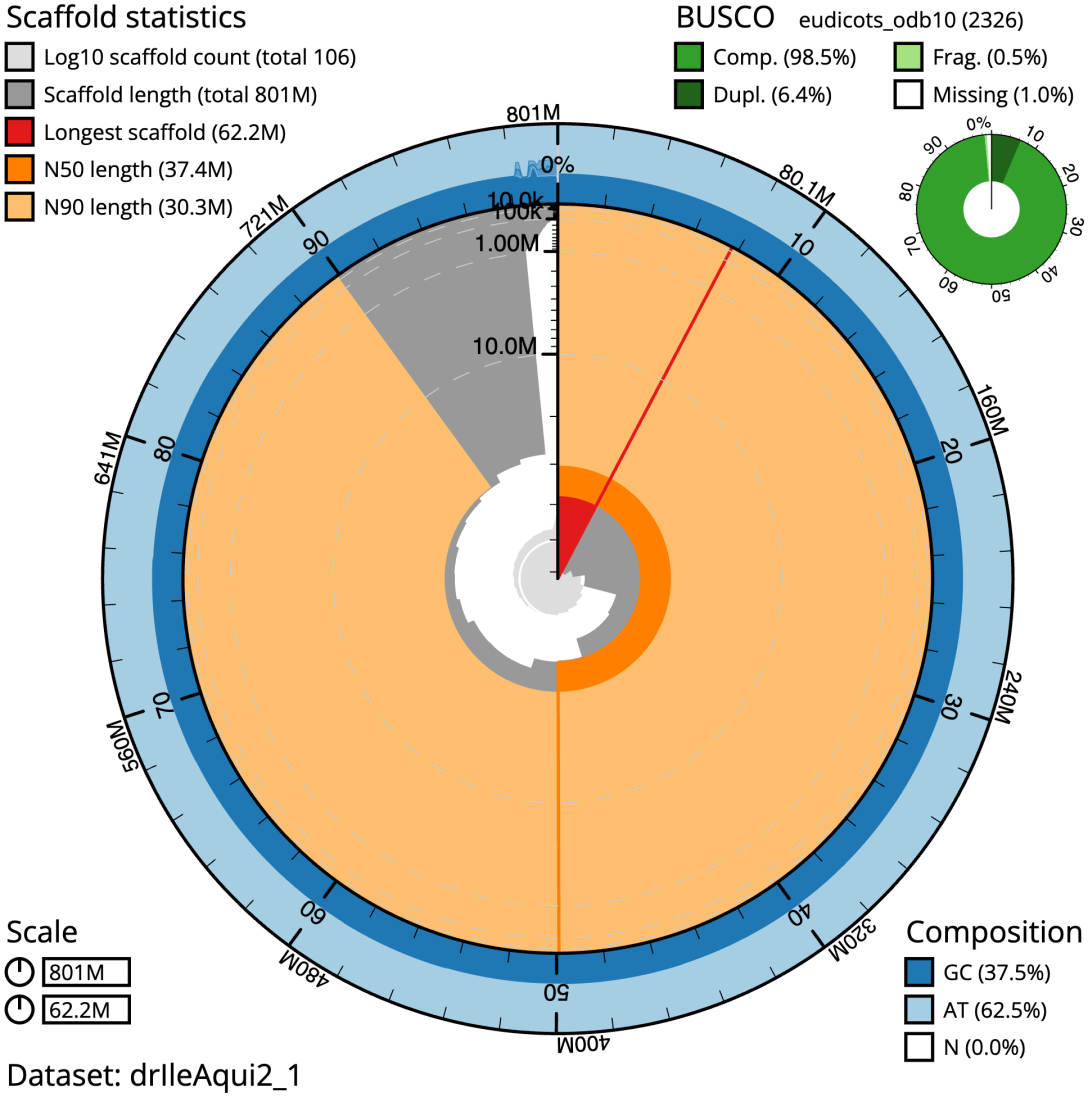
Genome assembly of *Ilex aquifolium*, drIleAqui2.1: metrics. The BlobToolKit Snailplot shows N50 metrics and BUSCO gene completeness. The main plot is divided into 1,000 size-ordered bins around the circumference with each bin representing 0.1% of the 800,663,922 bp assembly. The distribution of scaffold lengths is shown in dark grey with the plot radius scaled to the longest scaffold present in the assembly (62,197,019 bp, shown in red). Orange and pale-orange arcs show the N50 and N90 scaffold lengths (37,412,598 and 30,299,755 bp), respectively. The pale grey spiral shows the cumulative scaffold count on a log scale with white scale lines showing successive orders of magnitude. The blue and pale-blue area around the outside of the plot shows the distribution of GC, AT and N percentages in the same bins as the inner plot. A summary of complete, fragmented, duplicated and missing BUSCO genes in the eudicots_odb10 set is shown in the top right. An interactive version of this figure is available at https://blobtoolkit.genomehubs.org/view/drIleAqui2_1/dataset/drIleAqui2_1/snail.

**Figure 3.**
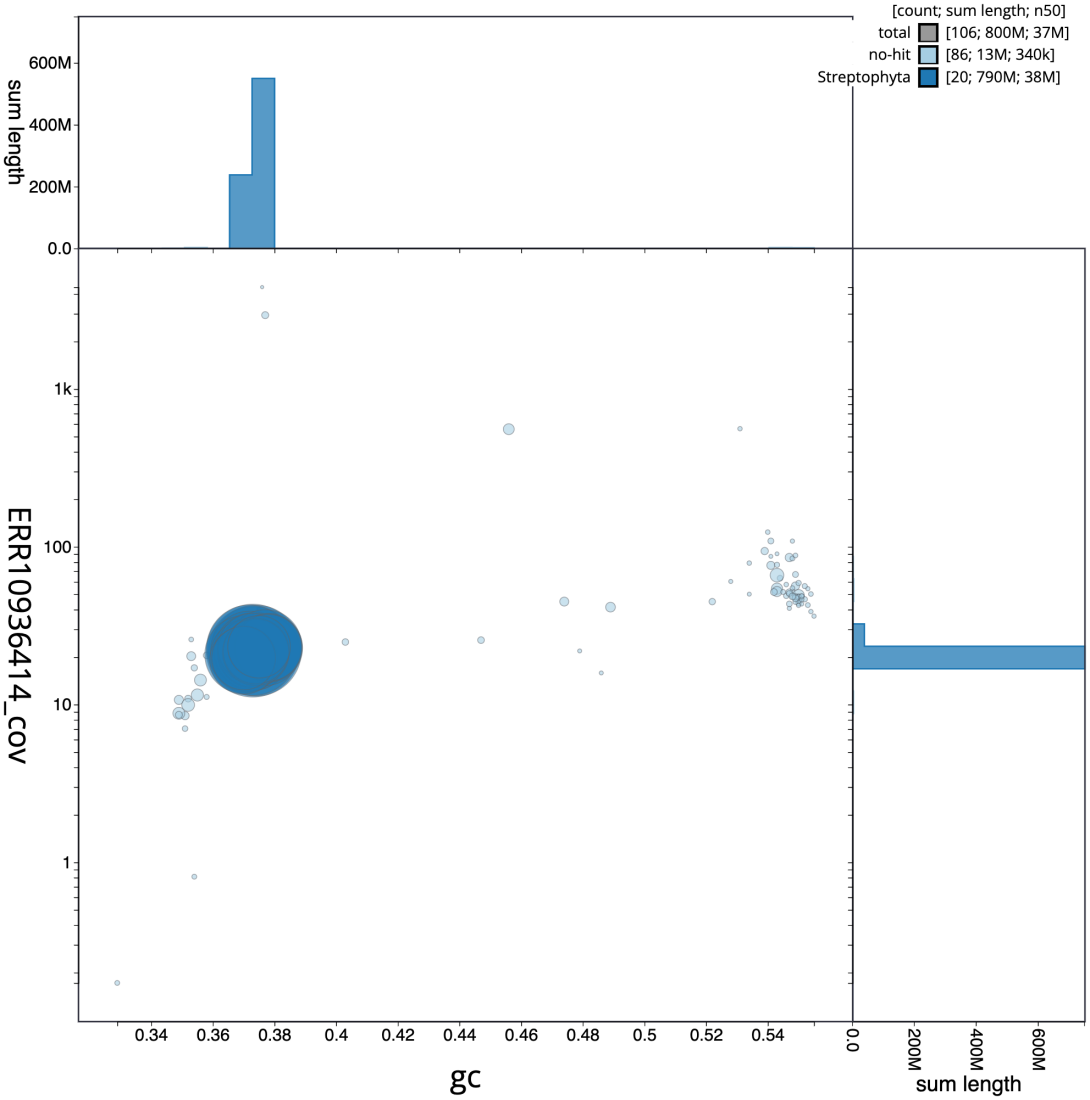
Genome assembly of *Ilex aquifolium*, drIleAqui2.1: BlobToolKit GC-coverage plot. Scaffolds are coloured by phylum. Circles are sized in proportion to scaffold length. Histograms show the distribution of scaffold length sum along each axis. An interactive version of this figure is available at https://blobtoolkit.genomehubs.org/view/drIleAqui2_1/dataset/drIleAqui2_1/blob.

**Figure 4.**
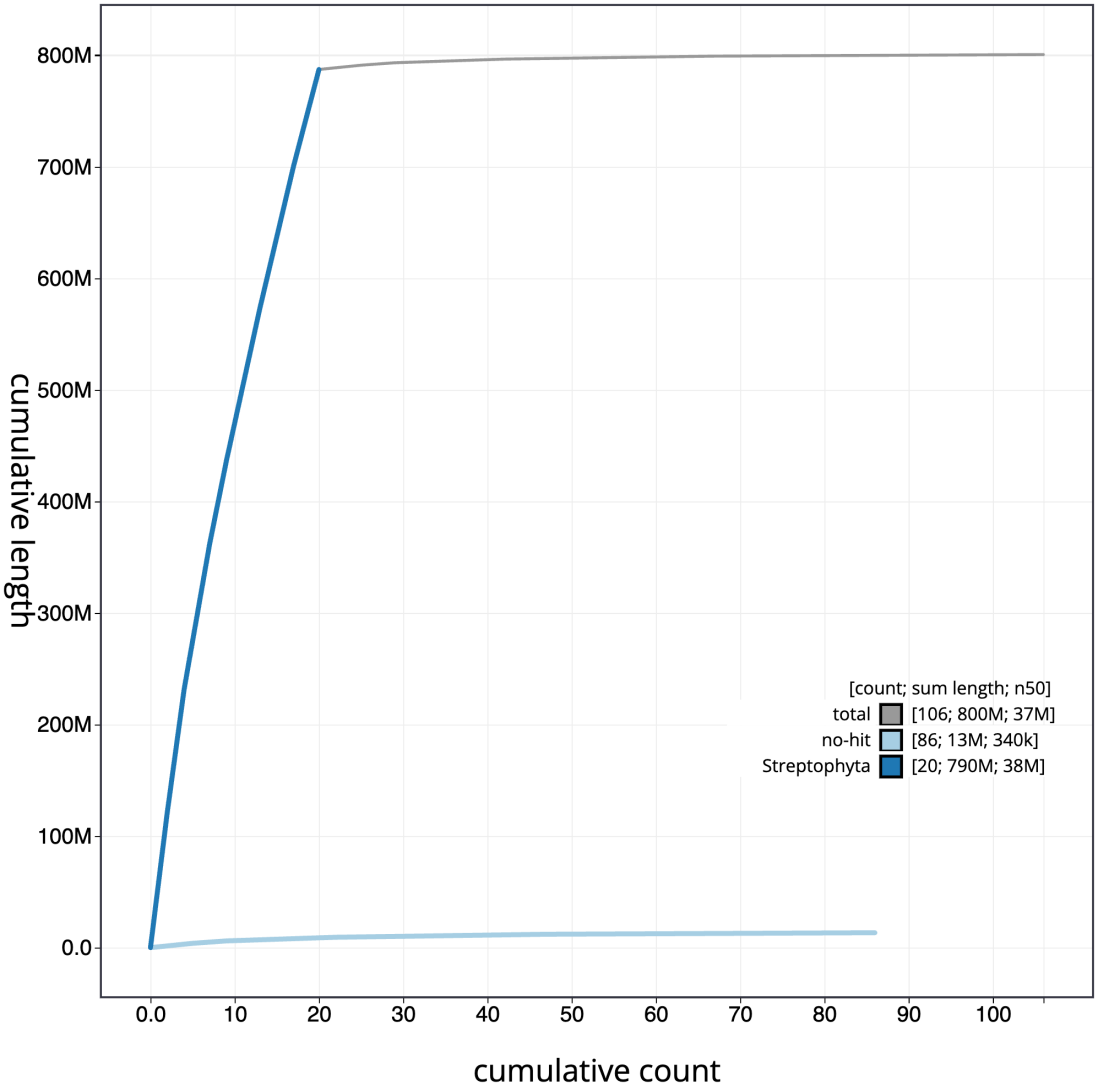
Genome assembly of *Ilex aquifolium*, drIleAqui2.1: BlobToolKit cumulative sequence plot. The grey line shows cumulative length for all scaffolds. Coloured lines show cumulative lengths of scaffolds assigned to each phylum using the buscogenes taxrule. An interactive version of this figure is available at https://blobtoolkit.genomehubs.org/view/drIleAqui2_1/dataset/drIleAqui2_1/cumulative.

**Figure 5.**
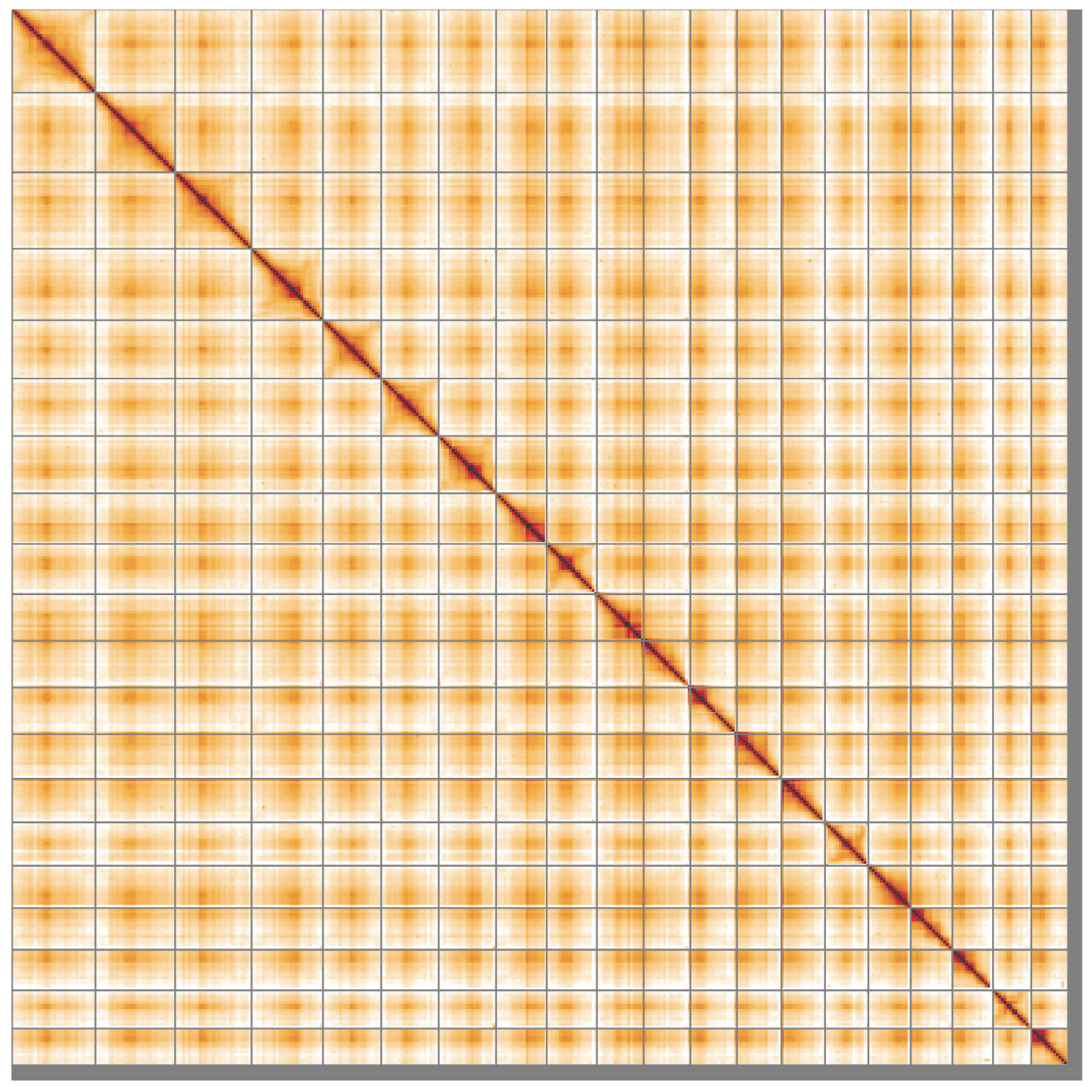
Genome assembly of *Ilex aquifolium*, drIleAqui2.1: Hi-C contact map of the drIleAqui2.1 assembly, visualised using HiGlass. Chromosomes are shown in order of size from left to right and top to bottom. An interactive version of this figure may be viewed at https://genome-note-higlass.tol.sanger.ac.uk/l/?d=NkyM_TiaRAqOF7SXZC8Kbg.

The final assembly has a total length of 800.0 Mb in 104 sequence scaffolds with a scaffold N50 of 37.4 Mb ( Table 1). The snailplot in Figure 2 provides a summary of the assembly statistics, while the distribution of assembly scaffolds on GC proportion and coverage is shown in Figure 3. The cumulative assembly plot in Figure 4 shows curves for subsets of scaffolds assigned to different phyla. Most (98.33%) of the assembly sequence was assigned to 20 chromosomal-level scaffolds. Chromosome-scale scaffolds confirmed by the Hi-C data are named in order of size ( Figure 5; Table 2). While not fully phased, the assembly deposited is of one haplotype. Contigs corresponding to the second haplotype have also been deposited. The mitochondrial and plastid genomes were also assembled and can be found as contigs within the multifasta file of the genome submission.

**Table 1.** Genome data for *Ilex aquifolium*, drIleAqui2.1.

Project accession data		
Assembly identifier	drIleAqui2.1	
Species	*Ilex aquifolium*	
Specimen	drIleAqui2	
NCBI taxonomy ID	4298	
BioProject	PRJEB60202	
BioSample ID	SAMEA7522524	
Isolate information	drIleAqui2, leaves (DNA and Hi-C sequencing)	
Assembly metrics *		*Benchmark*
Consensus quality (QV)	64.2	*≥ 50*
*k*-mer completeness	100.0%	*≥ 95%*
BUSCO **	C:98.5%[S:92.2%,D:6.4%], F:0.5%,M:1.0%,n:2,326	*C ≥ 95%*
Percentage of assembly mapped to chromosomes	98.33%	*≥ 95%*
Sex chromosomes	None	*localised homologous pairs*
Organelles	Mitochondrial genome: 538.43 kb Plastid genome: 157.52 kb	*complete single alleles*
Raw data accessions		
PacificBiosciences SEQUEL II	ERR10962203, ERR10934073, ERR10934075, ERR10934074	
Hi-C Illumina	ERR10936418	
Genome assembly		
Assembly accession	GCA_951799425.1	
*Accession of alternate haplotype*	GCA_951799435.1	
Span (Mb)	800.0	
Number of contigs	160	
Contig N50 length (Mb)	15.2	
Number of scaffolds	104	
Scaffold N50 length (Mb)	37.4	
Longest scaffold (Mb)	62.2	

* Assembly metric benchmarks are adapted from column VGP-2020 of “Table 1: Proposed standards and metrics for defining genome assembly quality” from Rhie *et al.* (2021). ** BUSCO scores based on the eudicots_odb10 BUSCO set using version 5.3.2. C = complete [S = single copy, D = duplicated], F = fragmented, M = missing, n = number of orthologues in comparison. A full set of BUSCO scores is available at https://blobtoolkit.genomehubs.org/view/drIleAqui2_1/dataset/drIleAqui2_1/busco.

**Table 2.** Chromosomal pseudomolecules in the genome assembly of *Ilex aquifolium*, drIleAqui2.

INSDC accession	Chromosome	Length (Mb)	GC%
OX637391.1	1	62.2	37.5
OX637392.1	2	59.41	37.5
OX637393.1	3	56.97	37.5
OX637394.1	4	53.23	37.0
OX637395.1	5	43.44	37.0
OX637396.1	6	42.84	37.0
OX637397.1	7	42.8	37.5
OX637398.1	8	37.73	37.5
OX637399.1	9	37.41	37.0
OX637400.1	10	34.85	37.5
OX637401.1	11	34.83	37.5
OX637402.1	12	34.75	37.5
OX637403.1	13	33.17	38.0
OX637404.1	14	32.47	38.0
OX637405.1	15	32.33	37.0
OX637406.1	16	31.56	38.0
OX637407.1	17	31.11	37.5
OX637408.1	18	30.3	37.5
OX637409.1	19	28.48	37.0
OX637410.1	20	27.43	37.5
OX637411.1	MT	0.54	45.5
OX637412.1	Pltd	0.16	37.5

The estimated Quality Value (QV) of the final assembly is 64.2 with *k*-mer completeness of 100.0%, and the assembly has a BUSCO v5.3.2 completeness of 98.5% (single = 92.2%, duplicated = 6.4%), using the eudicots_odb10 reference set ( *n* = 2,326).

Metadata for specimens, barcode results, spectra estimates, sequencing runs, contaminants and pre-curation assembly statistics are given at https://links.tol.sanger.ac.uk/species/4298.

## Methods

### Sample acquisition, genome size estimation and nucleic acid extraction

A specimen of *Ilex aquifolium* (specimen ID KDTOL10104, ToLID drIleAqui2) was picked by hand in Petersham Common, Richmond, Surrey, UK (latitude 51.45, longitude –0.30) on 2020-09-08. The specimen was collected and identified by Maarten J. M. Christenhusz (Independent) and frozen at –80 °C.

The genome size was estimated by flow cytometry using the fluorochrome propidium iodide and following the ‘one-step’ method as outlined in Pellicer *et al.* (2021). For this species, the General Purpose Buffer (GPB) supplemented with 3% PVP and 0.08% (v/v) beta-mercaptoethanol was used for isolation of nuclei ( Loureiro *et al.*, 2007), and the internal calibration standard was *Petroselinum crispum* ‘Champion Moss Curled’ with an assumed 1C-value of 2,200 Mb ( Obermayer *et al.*, 2002).

The workflow for high molecular weight (HMW) DNA extraction at the Wellcome Sanger Institute (WSI) includes a sequence of core procedures: sample preparation; sample homogenisation, DNA extraction, fragmentation, and clean-up. In sample preparation, the drIleAqui2 sample was weighed and dissected on dry ice ( Jay *et al.*, 2023). For sample homogenisation, leaf tissue was cryogenically disrupted using the Covaris cryoPREP ^®^ Automated Dry Pulverizer ( Narváez-Gómez *et al.*, 2023). HMW DNA was extracted using the Plant Organic gDNA Extraction method ( Jackson & Howard, 2023). HMW DNA was sheared into an average fragment size of 12–20 kb in a Megaruptor 3 system with speed setting 31 ( Bates *et al.*, 2023). Sheared DNA was purified by solid-phase reversible immobilisation ( Oatley *et al.*, 2023): in brief, the method employs a 1.8X ratio of AMPure PB beads to sample to eliminate shorter fragments and concentrate the DNA. The concentration of the sheared and purified DNA was assessed using a Nanodrop spectrophotometer and Qubit Fluorometer and Qubit dsDNA High Sensitivity Assay kit. Fragment size distribution was evaluated by running the sample on the FemtoPulse system.

Protocols developed by the WSI Tree of Life core laboratory are publicly available on protocols.io ( Denton *et al.*, 2023).

### Sequencing

Pacific Biosciences HiFi circular consensus DNA sequencing libraries were constructed according to the manufacturer’s instructions. DNA sequencing was performed by the Scientific Operations core at the WSI on a Pacific Biosciences SEQUEL II instrument. Hi-C data were also generated from leaf tissue of drIleAqui2 using the Arima2 kit and sequenced on the Illumina NovaSeq 6000 instrument.

### Genome assembly, curation and evaluation

Assembly was carried out with Hifiasm ( Cheng *et al.*, 2021) and haplotypic duplication was identified and removed with purge_dups ( Guan *et al.*, 2020). The assembly was then scaffolded with Hi-C data ( Rao *et al.*, 2014) using YaHS ( Zhou *et al.*, 2023). The assembly was checked for contamination and corrected using the gEVAL system ( Chow *et al.*, 2016) as described previously ( Howe *et al.*, 2021). Manual curation was performed using gEVAL, HiGlass ( Kerpedjiev *et al.*, 2018) and Pretext ( Harry, 2022). The organelle genomes were assembled using MitoHiFi ( Uliano-Silva *et al.*, 2023) and OATK ( Zhou, 2023).

A Hi-C map for the final assembly was produced using bwa-mem2 ( Vasimuddin *et al.*, 2019) in the Cooler file format ( Abdennur & Mirny, 2020). To assess the assembly metrics, the *k*-mer completeness and QV consensus quality values were calculated in Merqury ( Rhie *et al.*, 2020). This work was done using Nextflow ( Di Tommaso *et al.*, 2017) DSL2 pipelines “sanger-tol/readmapping” ( Surana *et al.*, 2023a) and “sanger-tol/genomenote” ( Surana *et al.*, 2023b). The genome was analysed within the BlobToolKit environment ( Challis *et al.*, 2020) and BUSCO scores ( Manni *et al.*, 2021; Simão *et al.*, 2015) were calculated.

Table 3 contains a list of relevant software tool versions and sources.

**Table 3.** Software tools: versions and sources.

Software tool	Version	Source
BlobToolKit	4.1.7	https://github.com/blobtoolkit/blobtoolkit
BUSCO	5.3.2	https://gitlab.com/ezlab/busco
gEVAL	-	https://geval.org.uk/
Hifiasm	0.16.1-r375	https://github.com/chhylp123/hifiasm
HiGlass	1.11.6	https://github.com/higlass/higlass
Merqury	MerquryFK	https://github.com/thegenemyers/MERQURY.FK
MitoHiFi	2	https://github.com/marcelauliano/MitoHiFi
OATK	0.1	https://github.com/c-zhou/oatk
PretextView	0.2	https://github.com/wtsi-hpag/PretextView
purge_dups	1.2.3	https://github.com/dfguan/purge_dups
sanger-tol/genomenote	v1.0	https://github.com/sanger-tol/genomenote
sanger-tol/readmapping	1.1.0	https://github.com/sanger-tol/readmapping/tree/1.1.0
YaHS	1.2a	https://github.com/c-zhou/yahs

### Wellcome Sanger Institute – Legal and Governance

The materials that have contributed to this genome note have been supplied by a Darwin Tree of Life Partner. The submission of materials by a Darwin Tree of Life Partner is subject to the **‘Darwin Tree of Life Project Sampling Code of Practice’**, which can be found in full on the Darwin Tree of Life website here. By agreeing with and signing up to the Sampling Code of Practice, the Darwin Tree of Life Partner agrees they will meet the legal and ethical requirements and standards set out within this document in respect of all samples acquired for, and supplied to, the Darwin Tree of Life Project. 

Further, the Wellcome Sanger Institute employs a process whereby due diligence is carried out proportionate to the nature of the materials themselves, and the circumstances under which they have been/are to be collected and provided for use. The purpose of this is to address and mitigate any potential legal and/or ethical implications of receipt and use of the materials as part of the research project, and to ensure that in doing so we align with best practice wherever possible. The overarching areas of consideration are:

•   Ethical review of provenance and sourcing of the material

•   Legality of collection, transfer and use (national and international) 

Each transfer of samples is further undertaken according to a Research Collaboration Agreement or Material Transfer Agreement entered into by the Darwin Tree of Life Partner, Genome Research Limited (operating as the Wellcome Sanger Institute), and in some circumstances other Darwin Tree of Life collaborators.

## Data Availability

European Nucleotide Archive: *Ilex aquifolium* (English holly). Accession number PRJEB60202; https://identifiers.org/ena.embl/PRJEB60202 ( Wellcome Sanger Institute, 2023). The genome sequence is released openly for reuse. The *Ilex aquifolium* genome sequencing initiative is part of the Darwin Tree of Life (DToL) project. All raw sequence data and the assembly have been deposited in INSDC databases. The genome will be annotated using available RNA-Seq data and presented through the Ensembl pipeline at the European Bioinformatics Institute. Raw data and assembly accession identifiers are reported in Table 1.

## Author information

Members of the Royal Botanic Gardens Kew Genome Acquisition Lab are listed here: https://doi.org/10.5281/zenodo.4786680.

Members of the Plant Genome Sizing collective are listed here: https://doi.org/10.5281/zenodo.7994306.

Members of the Darwin Tree of Life Barcoding collective are listed here: https://doi.org/10.5281/zenodo.4893703.

Members of the Wellcome Sanger Institute Tree of Life Management, Samples and Laboratory team are listed here: https://doi.org/10.5281/zenodo.10066175.

Members of Wellcome Sanger Institute Scientific Operations: Sequencing Operations are listed here: https://doi.org/10.5281/zenodo.10043364.

Members of the Wellcome Sanger Institute Tree of Life Core Informatics team are listed here: https://doi.org/10.5281/zenodo.10066637.

Members of the Tree of Life Core Informatics collective are listed here: https://doi.org/10.5281/zenodo.5013541.

Members of the Darwin Tree of Life Consortium are listed here: https://doi.org/10.5281/zenodo.4783558.
