# Supplementary material for: The genome sequence of the English holly, Ilex aquifolium L. (Aquifoliaceae)
Source: Wellcome Open Res. 2024 Jan 3;9:1. [Version 1] doi: 10.12688/wellcomeopenres.20748.1 (PMC11109700; doi:10.12688/wellcomeopenres.20748.1)
Supplement: Peer review history

# Reviewer report: Reviewer response for version 1

**DOI:** 10.21956/wellcomeopenres.22960.r80518

**Published:** 2024-05-13

**Authors:** Paola Gaiero

**Affiliations:** 1 Universidad de la República, Montevideo, Uruguay

This dataset reports the genome sequencing and assembly of a highly emblematic and popular plant, the English holly. The significant cultural value and traditional knowledge associated to this plant, together with all the links with landmark dates for western civilization, make it a very suitable species to be included in the Tree of Life project and justifies the rationale behind creating this dataset. 

 The genome was sequenced with the latest technologies applied for plant genomes and the scaffolding was properly achieved to the chromosome level using high quality Hi-C data as presented in the contact map in Figure 5. The assembly of one haplotype is presented and the contigs for the alternative haplotyped are also provided. Organelle genomes are also assembled and presented. All methods are sound, robust and clearly presented to a detail that would allow replication. Access to all the software and datasets is easily provided. 

 A very informative snailplot is used to present the different features of this genome assembly. Metrics in this plot are compared to standards in table 1 and are all highly satisfactory. Although I understand that it is a dataset report and therefore all the metrics and features of this genome are to be presented and exploited, I am not entirely sure about the relevance of Figures 3 and 4. If possible, I would suggest the authors to include an explanation or interpretation of why the genome assembly manages to cover 800 Mbp out of the estimated genome size of 1010 Mbp. 

 To sum up, this is a sound and robust dataset, obtaining a high quality, chromosome-scale genome assembly of the emblematic English holly, which makes publicly available a full haplotype together with the contigs for the alternative haplotype and the chloroplast and mitochondrial full genome assemblies. It is undoubtfully a useful contribution to the plant genomics community and to the Tree of life project. 

Are sufficient details of methods and materials provided to allow replication by others?

Yes

Is the rationale for creating the dataset(s) clearly described?

Yes

Are the datasets clearly presented in a useable and accessible format?

Yes

Are the protocols appropriate and is the work technically sound?

Yes

Reviewer Expertise:

Comparative plant genomics

I confirm that I have read this submission and believe that I have an appropriate level of expertise to confirm that it is of an acceptable scientific standard.

# Reviewer report: Reviewer response for version 1

**DOI:** 10.21956/wellcomeopenres.22960.r80521

**Published:** 2024-05-09

**Authors:** Xin Yao

**Affiliations:** 1 Center for Integrative Conservation, Xishuangbanna Tropical Botanical Garden, University of the Chinese Academy of Sciences, Beijing, Beijing, China

Species *Ilex aquifolium* is an important species in terms of its values of culture and ecology. The chromosome-scale genome of this species will be definitely helpful to future studies of its ecology, evolution, breeding, and so on. However, the manuscript could improve.

 Firstly, the background was not well structured. The authors used eight paragraphs to introduce the morphology, culture, and utilization of *I. aquifolium*. All these contents could merge into one or two paragraphs. Yet, the chromosome-scale genome of this species would be more directly beneficial to its ecology, evolution, and breeding. So, the background could talk a little more about how these aspects need a chromosome-scale genome. Try to keep the whole background concise and readable.

 Secondly, the methods section was not very clear. Did the authors only use the sequence data produced by the third-generation sequencer and Hi-C? What were the sequencing depths of the third-generation sequencing and Hi-C sequencing? Ideally, authors should make a genome comparison between the *I. aquifolium* genome and other published Ilex genomes. Hope that the annotations of this I. aquifolium genome could be published soon

Are sufficient details of methods and materials provided to allow replication by others?

No

Is the rationale for creating the dataset(s) clearly described?

Partly

Are the datasets clearly presented in a useable and accessible format?

Yes

Are the protocols appropriate and is the work technically sound?

Yes

Reviewer Expertise:

Phylogenetics, biogeography, and population genomics.

I confirm that I have read this submission and believe that I have an appropriate level of expertise to confirm that it is of an acceptable scientific standard, however I have significant reservations, as outlined above.

# Reviewer report: Reviewer response for version 1

**DOI:** 10.21956/wellcomeopenres.22960.r80522

**Published:** 2024-05-06

**Authors:** Jian-Feng Mao

**Affiliations:** 1 Umeå University, Umeå, Sweden

This is a report on one genome sequence data release from Darwin Tree of Life Consortium. The whole project is well structured and managed. Selection of the targeted species, the sampling, sequencing, assembly and report generation are all in well executed under a very much clearly established protocol. 

 This data report is well prepared, and the genome quality is very good. I will have only simple concerns to discuss with the authors.

 1. How about gaps? How many of them? Their distribution? 

 2. Heterozygosity? It could be calculated from K-mer profile. It is important in some way.

 3. You did not provide genome annotation (gene and repeat, centromere, telomere etc.). Why?

Are sufficient details of methods and materials provided to allow replication by others?

Partly

Is the rationale for creating the dataset(s) clearly described?

Yes

Are the datasets clearly presented in a useable and accessible format?

Partly

Are the protocols appropriate and is the work technically sound?

Yes

Reviewer Expertise:

Plant genomics, bioinformatics, genetics and breeding, evolution.

I confirm that I have read this submission and believe that I have an appropriate level of expertise to confirm that it is of an acceptable scientific standard, however I have significant reservations, as outlined above.
